# Efficient Defect Detection of Rotating Goods under the Background of Intelligent Retail

**DOI:** 10.3390/s24020467

**Published:** 2024-01-12

**Authors:** Zhengming Hu, Xuepeng Zeng, Kai Xie, Chang Wen, Jianbiao He, Wei Zhang

**Affiliations:** 1 School of Electronic Information and Electrical Engineering, Yangtze University, Jingzhou 434023, China; 2022001407@yangtzeu.edu.cn (Z.H.); xuepeng_zeng.st@yangtzeu.edu.cn (X.Z.); 2 School of Computer Science, Yangtze University, Jingzhou 434023, China; 400100@yangtzeu.edu.cn; 3 School of Computer Science, Central South University, Changsha 410083, China; jbhe@mail.csu.edu.cn; 4 School of Electronic Information, Central South University, Changsha 410083, China; csuzwzbn@csu.edu.cn

**Keywords:** anomaly detection, few-shot learning, memory repository, dynamic visual vending

## Abstract

Dynamic visual vending machines are rapidly growing in popularity, offering convenience and speed to customers. However, there is a prevalent issue with consumers damaging goods and then returning them to the machine, severely affecting business interests. This paper addresses the issue from the standpoint of defect detection. Although existing industrial defect detection algorithms, such as PatchCore, perform well, they face challenges, including handling goods in various orientations, detection speeds that do not meet real-time monitoring requirements, and complex backgrounds that hinder detection accuracy. These challenges hinder their application in dynamic vending environments. It is crucial to note that efficient visual features play a vital role in memory banks, yet current memory repositories for industrial inspection algorithms do not adequately address the problem of location-specific feature redundancy. To tackle these issues, this paper introduces a novel defect detection algorithm for goods using adaptive subsampling and partitioned memory banks. Firstly, Grad-CAM is utilized to extract deep features, which, in combination with shallow features, mitigate the impact of complex backgrounds on detection accuracy. Next, graph convolutional networks extract rotationally invariant features. The adaptive subsampling partitioned memory bank is then employed to store features of non-defective goods, which reduces memory consumption and enhances training speed. Experimental results on the MVTec AD dataset demonstrate that the proposed algorithm achieves a marked improvement in detection speed while maintaining accuracy that is comparable to state-of-the-art models.

## 1. Introduction

The contemporary retail sector is in the midst of a dynamic shift towards digitalization, led by the advent of intelligent, dynamic visual vending machines. These advanced machines, now a common sight, owe their proliferation to the synergistic advancements in mobile payments, AI, and cloud computing, which have collectively accelerated the unmanned retail industry [1]. Despite this progress, the prevalence of damaged merchandise represents a significant impediment to their full-scale adoption. Conventional methods of product inspection are no longer viable options, as they cannot adequately cater to the demands for speed, efficiency, or precision, thereby necessitating a dependable mechanism for damage assessment.

Interest in deep visual inspection technology, especially within AI for detecting flaws like scratches, stains, or absences on product surfaces, has surged considerably. This development suggests that methodologies designed for industrial defect detection could be adapted and refined to address the unique challenges presented by dynamic visual vending machines.

The current landscape of industrial anomaly detection is characterized by two primary issues: data acquisition and training efficiency. Dependence on comprehensive datasets for training makes gathering sufficient data burdensome. Although meta-learning approaches such as those described by Huang et al. [2] facilitate learning from fewer samples, extensive data collection is still a prerequisite. Simultaneously, introducing new product models within actual production environments is often sluggish despite efforts to expedite the process through meta-learning.

A majority of defect detection strategies based on feature similarity employ a memory repository, with the PatchCore model [3] receiving recognition for its efficacy as it leverages ResNet [4] to extract image features. Yet, due to the non-rotational invariance [5] of CNN-extracted features, these repositories amass a plethora of redundant features, primarily composed of rotationally varied instances of identical patch structures. For smart vending machines, where merchandise is subject to varying angles and rotations, the extraction of rotationally invariant features is of paramount importance.

Recent studies by Guo et al. have emphasized the potential of GNNs in effectively extracting non-linear structural features [6,7,8], arguing for their superiority over CNNs in obtaining rotationally invariant features. This insight led to the development of the Augmentation +PatchCore approach, addressing the challenges of rotational invariance, and subsequently, the GraphCore model [9], which enables rapid model training and maintains competitive levels of anomaly detection with a limited dataset of normal samples.

In the present work, we address the intricacies involved in detecting damage within smart vending machine inventories. These complexities include diverse product orientations by users, intricate backdrop scenarios within consumer settings, and practical constraints related to computational and storage capacities when applying feature embedding-based detection models.

Confronted with these challenges, we propose the application of a GNN to extract rotation-invariant features of the products. In a novel departure from the GraphCore framework, we advance a combined CNN+GNN methodology to distill features. Initially, a pre-trained CNN network extracts foundational image attributes, followed by a GNN that remodels these features into graph forms, subsequently engaging in graph convolution processing. Our approach also includes comprehensive measures to curtail background discrepancies from influencing detection, with a detailed outline presented in Section 2.

In pursuit of managing extensive vector features with greater efficacy and increasing the speed of detection, we put forth the concept of Adaptive Subsampled Partition Memory (ASPM). The ASPM model is predicated on the idea that feature vectors from corresponding image patches tend to exhibit similarity. By segregating features by storing them as distinct patches, this method mitigates the need for up-sampling and minimizes computational overhead during the comparison stage. Furthermore, our introduction of adaptive core sampling refines the inference rate by retaining only the most significant vectors, thus reducing the quantum of vectors archived.

The innovative methods described in this paper include the following:

1. Designing a CNN+GNN feature extraction approach to solve the feature extraction problem of retail goods with rich information;
2. Utilizing Grad-CAM technology to reduce the impact of complex environments on the goods to be inspected;
3. Designing an adaptive subsampling memory for efficient feature storage, which reduces storage requirements and speeds up computation.

These approaches to solving the problem of detecting defects in goods in dynamic visual vending machines help to advance the current development of defect detection and have a promising future.

## 2. Related Work

Anomaly detection is an increasingly important research domain in computer vision, with applications in industrial inspection, quality control, and more. Recent progress has leveraged deep learning to learn effective representations of nominal data. During inference, anomaly scores are computed based on feature similarity-based approaches. The existing literature underscores challenges associated with pixel-level comparisons, such as misalignment and style variations during the reconstruction process. Researchers have shifted their focus towards feature space, leveraging deep neural networks for robust detection.

### 2.1. Deep One-Class Classification

One prominent approach is deep one-class classification, which constructs a high-quality feature space using deep neural networks [10]. These models aim to extract discriminative feature embeddings, enhancing robustness and tolerance to noise. Early hybrid methods combined deep learning with traditional approaches [11], demonstrating improved performance. Advanced techniques, like OC-NN [10] and Deep SVDD [12], address limitations, such as the need for manually specified feature centers. However, these methods are not without challenges, including the need for manual feature center specification, assumptions about the distribution of normal samples, and potential model degradation issues. Strategies for mitigating these challenges involve optimizing the feature extraction process and introducing modifications to enhance detection capabilities [13].

### 2.2. Feature Distance Metrics

Feature distance metric methods eliminate the need for optimizing boundaries in feature space. These approaches rely on appropriate feature representations obtained from pre-trained models, either through vector-based or distribution-based metrics. Matching normal templates to test samples directly or modeling normal sample distributions using Gaussian distributions are common strategies [14,15]. While methods like SPADE [14] and NF-based models [16] demonstrate effectiveness, they may have limitations in handling diverse datasets and variations. Techniques employing knowledge distillation, such as teacher-student frameworks [17], have proven successful in achieving pixel-level anomaly segmentation. Still, challenges persist in terms of fixed receptive fields and increased model complexity.

### 2.3. Unsupervised and Fully Unsupervised Approaches

Recent advances in unsupervised and fully unsupervised approaches showcase promising results. TrustMAE [18] employs a trust region-based update mechanism, preventing anomaly features from contaminating memory. Another method, based on clustering principles [19], assumes higher variance in defect samples and iteratively excludes them during training. Fully unsupervised settings, where no labeled data are available, have gained attention due to their potential for cost-effective application in highly imbalanced industrial datasets. The exploration of completely unsupervised settings, exemplified by TrustMAE [18] and clustering-based techniques [19], demonstrates the ongoing development in this field.

In order to achieve good defect detection results, peers have made a lot of explorations. Defard et al. presented PaDiM [20] for distribution modeling of image patches using pre-trained CNN and Gaussian models. This approach achieved superior detection and localization on the MVTec AD and STC datasets while remaining efficient for industrial use. Cohen and Hoshen extended the deep SVDD [12] algorithm to enable patch-level learning under self-supervision, improving anomaly detection and segmentation accuracy on MVTec AD. Cohen and Hoshen devised SPADE [14] for anomaly segmentation by aligning images using multi-resolution correspondences between anomalous and normal examples. SPADE excelled at unsupervised detection and masking without training. Roth et al. introduced PatchCore [3] to address cold-start defect detection by memorizing a representative bank of nominal patch features. PatchCore reduced anomaly detection error by 50% on MVTec AD while maintaining competitive inference latency. Yang et al. developed MemSeg [21], a semi-supervised memory-based network performing surface defect segmentation. MemSeg incorporates simulated anomalies and normal memory patterns to explicitly contrast normal and abnormal. It achieved top performance on the MVTec AD dataset for detection and localization.

## 3. Methodology

**Figure 1:**
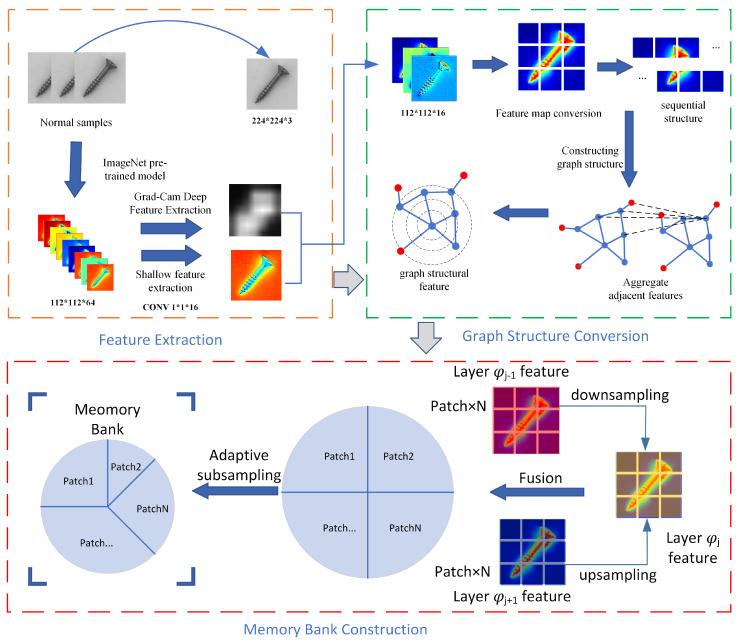
Algorithmic process.

Figure 1 is the flowchart of the complete algorithm in this paper, which contains three parts: image spatial feature extraction, image rotation invariant feature extraction, and memory repository construction. (1) The underlying features of the image are extracted by using the first three layers of a network pre-trained on a large dataset (ImageNet [22]), and spatial attention maps are obtained using the Grad-CAM [23]. (2) Then, the previous underlying features are converted to graph structure using a GCN [24] network to extract the rotation-invariant features of the image. (3) Finally, the features are partitioned for storage and subsampled to create the final memory repository, which contains features of normal goods.

### 3.1. Feature Extraction

#### 3.1.1. Spatial Feature Extraction

GraphCore uses GNN to extract rotationally invariant features, which can be a good solution to the problem of defect detection in rotating industrial products. However, most of the detected objects in the industrial inspection dataset are isomorphic, and unlike the isomorphic objects in the industrial inspection dataset, the retail goods not only have different shapes when they are picked up but also have rich information about the packaging of the goods. If only the GNN is used to extract the rotationally invariant features, it will lead to insufficient feature extraction, and the GNN pays more attention to extracting the dependencies between structures and global features, so we choose to extract the spatial features at once with CNN, then transform this spatial feature into N nodes, then use the GNN to extract the relationships between nodes at once. Therefore, we design a hybrid network structure to utilize the advantages of both, as follows:

(1) First, the features of normal samples are extracted using the network pre-trained on ImageNet [22], and the features of the first three layers are selected as the image features to be input to the subsequent feature extraction network. The reason why the first few layers are selected here is that they have strong underlying features (contours, edges, color, texture, and shape features), which are more reflective of the image content, while the deeper features in the later layers are biased toward classification tasks with stronger semantic information;

(2) Using Grad-CAM [23], we parse the category response activation of the pre-trained model (the output of the last convolutional layer) to produce a spatial attention map that highlights the salient areas within the image requiring heightened focus.

(1) $$P=f_{grad-cam}\left(X;\theta \right),$$

where P denotes attention map; $X\in R^{H\times W\times C}$ denotes the input image; and H,W,C are the height, width, and number of channels, respectively. θ denotes the parameters of the image classification model. $f_{grad-cam}\left(\cdot \right)$ is the computation process of global mean pooling and back propagation in Grad-CAM; 

(3) To further hone the spatial attention map, a non-linear mapping function is applied. This function recalibrates the attention value distribution, intensifying the focus on areas that exhibit high responses and thereby enhancing signal detection in critical zones.

(2) $$x_{i}=\left\{\begin{array}{l} 0.1\times x_{i},x_{i}<20\\ 1.1\times x_{i},x_{i}>=20 \end{array}\right.,$$

where $x_{i}$ represents each value in the P . We found experimentally that in our self-created goods breakage dataset, after the sample images had been processed with Grad-CAM to extract attention heat maps (with pixel values between 0 and 255), the vast majority of the background pixels exhibited values less than 20, while the pixels representing the main parts of the goods to be detected typically had values greater than 20. Therefore, we set the threshold at 20, which is an empirical value that may need slight adjustments when applied to other datasets;

(4) The optimized attention map is dot-multiplied with the feature map of step 1 to achieve feature enhancement of the target region of the subject of the input image while suppressing background noise.

(3) $$Z=g(X,P)=vec(P)⊙X_{sl},$$

where $Z\in R^{h\times w\times c}$ is the output augmented feature map of dot product. $X_{sl}\in R^{h\times w\times c}$ denotes the feature map of a shallow layer in the model. $vec\left(\cdot \right)$ denotes the reshaping of the attention map into vectors. ⊙ denotes the dot product operation. $g\left(\cdot \right)$ completes the dot product computation of the whole feature augmentation.

**Figure 2:**
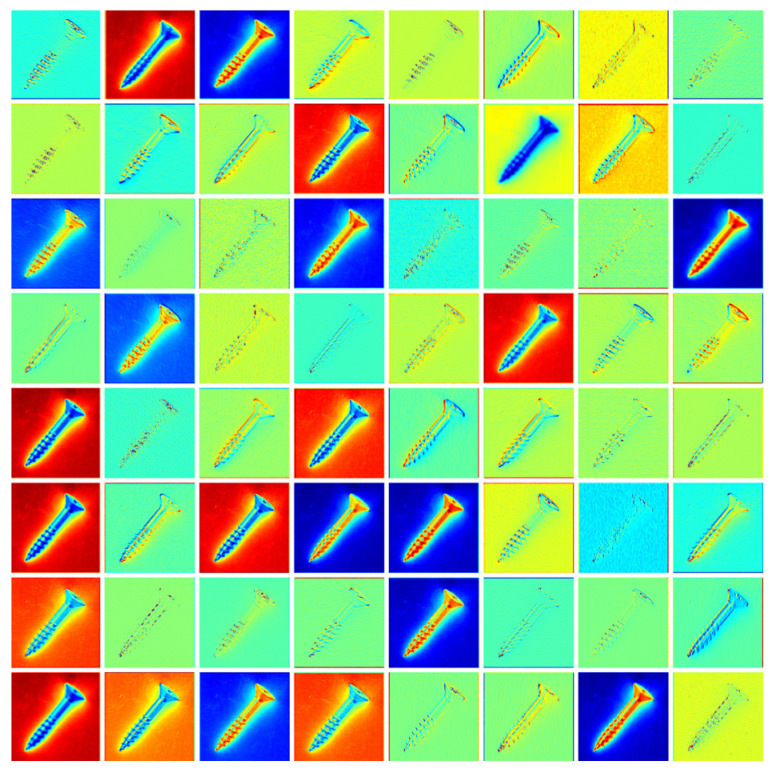
Layer 1 features extracted using ResNet50.

The features of the first three layers are processed and passed through 1 × 1 convolutions with 16 channels, respectively, so that they all become 16-channel feature maps, interacting and integrating the information between different channels, thus improving the ability of feature expression without changing the spatial structure of the image. Figure 2 shows the first layer feature map of ResNet50 [25].

#### 3.1.2. Rotationally Invariant Feature Extraction

**Figure 3:**
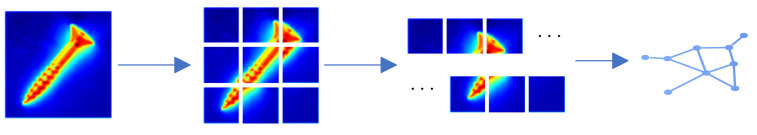
Extracting rotationally invariant features.

Here, we use the same method as GraphCore to construct the graph structure and employ GCN to extract the rotation-invariant features; As shown in Figure 3, what is displayed is the process of transforming spatial features into a graph structure. however, the difference is that we convert the feature map, which has been extracted using 1 × 1 convolutional layers with 16 channels, into a graph structure. Therefore, the dimensions of a normal sample image are H × W × 16. The other methods employed are consistent with those of GraphCore. In the feature extraction phase, which is set to GCN, the features of each node are aggregated by exchanging information with neighboring nodes. The specific feature extraction process is as follows:

(4) $$G^{\prime }=F\left(G,w\right)=Update\left(Aggregate\left(G,W_{aggregate}\right),W_{aggregate}\right),$$

where $W_{aggregate}$ and $W_{update}$ denote the weights of aggregation and update operations. They can both be optimized in an end-to-end manner.

### 3.2. Memory Repository Module Construction

Given that rotation-invariant features of normal goods have been extracted using a graph convolutional network (GCN), these features are reliably consistent across different rotations of similar goods. This consistency is reflected in the feature matrix, where the features from corresponding parts of different goods are stored at the same index, regardless of the goods’ orientation. Utilizing this principle, ASPM parcels the feature map into segmented patches during training and stores the embedded features of each segment discretely. This approach contrasts with conventional methods, where the entirety of an image’s features is stored collectively.

Existing anomaly detection methods like PatchCore [3] and PaDiM [20] typically extract multiple feature maps from an image, each representing different semantic layers. Simply concatenating these feature maps creates a comprehensive representation of the image. However, this practice can become inefficient and computationally burdensome due to the diminishing size of feature maps as you go deeper into the network, often necessitating an upsampling process to equalize the sizes for concatenation.

To optimize this process and reduce unnecessary computational overhead, a slight modification is introduced to the traditional concatenation technique. The modification involves designating the second layer of the pre-trained ImageNet network as the foundation or base layer. Then, the other two layers (from the network’s first three) are either downsampled [22] or upsampled [26] as appropriate to align with the base layer’s dimensions before being merged along the channel axis. This ensures a more efficient and coherent feature representation without imposing the costs associated with upsampling smaller-resolution feature maps.

This strategic approach to feature processing enables a more streamlined and potentially more effective anomaly detection framework, tailored for industrial scenarios where object rotation and complex features are common challenges. Through this processing, storage costs are reduced. For the feature map of the non-baseline layer, the sampling operation is defined as follows:

(5) $$S\left(F_{1}\right)=T\left(F_{1},p_{1},s_{1}\right),$$

where $F_{1}$ is the lth layer feature map, T denotes the sampling transform, $p_{1}$ is the sampling pattern, and $s_{1}$ is the sampling scale. The final multilayer feature map is represented in series as follows:

(6) $$F_{fusion}=Concat\left(S\left(F_{1}\right),S\left(F_{2}\right),\ldots ,S\left(F_{l}\right)\right)$$

The feature block is decomposed into $N_{p}$ lattice blocks, and each patch is represented by a feature vector of fixed length $N_{v}$ ( $N_{v}$ corresponds to the number of channels after concatenation), where $\{v_{1}^{i},v_{2}^{i},\ldots ,v_{N_{v}}^{i}\}\in V_{i}$ . These feature vectors are held by the patch-level memory $m_{i}$ , which holds the feature vectors of all $N_{A}$ images at a particular location *i*, $\left\{v_{i}^{1},v_{i}^{2},\ldots ,v_{i}^{N}\right\}\in m_{i}$ . $N_{A}$ is the total number of normal images in total. All memory locations are collected to form a patch-wise memory bank, $\left\{m_{1},m_{2},\ldots ,m_{N_{p}}\right\}\in M$ , where each $m_{i}$ denotes $N_{A}$ specific region of training images. Store the local perceptual feature vector $V_{i}$ of the normal image in the correct location of the patch memory $m_{i}$ . The total repository of all features in the set is defined as follows:

(7) $$M\in R^{N_{p}\times N_{A}\times N_{v}}$$

### 3.3. Adaptive Sampling

**Figure 4:**
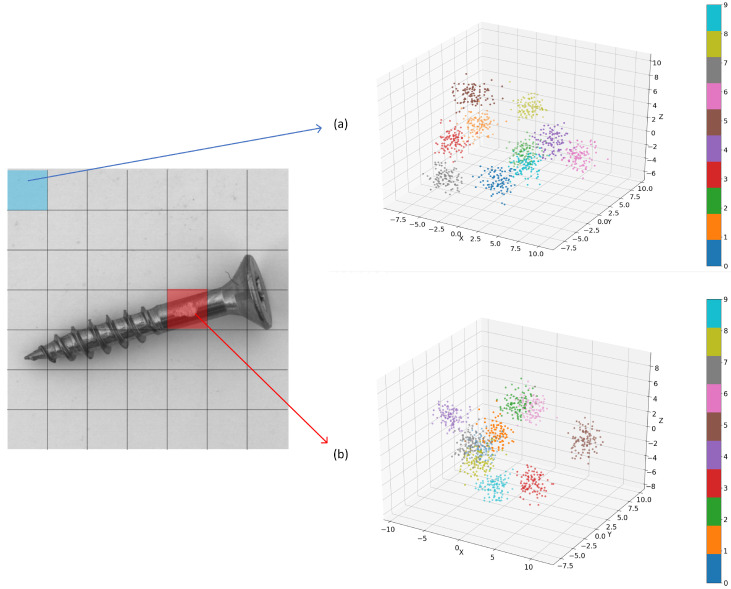
PCA of feature vectors with different patches. (**a**) is the PCA dimensionality reduction of the background section’s features, (**b**) is the PCA dimensionality reduction of the screw body section’s features.

Figure 4 shows the visualization after clustering the feature vectors of different patches of screws (k value of 10 for dimensionality reduction) and then performing PCA dimensionality reduction.

Figure 4a,b show that the distribution of feature vectors is different for different patches, and although the total number of feature vectors is the same for different patches, the number of key vectors is different. Therefore, it is not reasonable to simply use greedy core sampling [20] to sample the whole memory bank, so each patch should be sampled separately to retain the most representative feature vectors and remove the redundant ones.

In order to improve the inference speed while reducing the storage pressure, we propose patch-based adaptive core sampling for different patch feature sampling. First, the vectors in each patch are clustered (i.e., *K*-means clustering is performed on $m_{i}$ ), with the initial number of clusters *K* set to 10. Next, the center samples of these 10 clusters $V_{ik}$ are identified.

(8) $$\mu_{k}=\frac{1}{N_{K}}\sum_{f\in C_{k}}f,$$

where $C_{k}$ is the kth cluster, f is a sample point in that cluster, and $N_{K}$ is the number of sample points in cluster $C_{k}$ .

(9) $${V_{ik}}^{j}=\arg\min_{f\in C_{k}}dist(f,\mu_{k}),$$

where dist is the distance calculation function, the Euclidean distance is used. Then, the distance ${d_{i}}^{q-v}$ ( q,v∈[1,k] ) between each two centers is calculated separately. The distance calculation formula is as follows:

(10) $${d_{i}}^{q-v}=\frac{2}{0.5+\exp(-\left\|{V_{ik}}^{q}\right.-\left.{V_{ik}}^{v}\right\|)},$$

When assessing feature distances against a predefined threshold $D_{th}$ , our methodology dictates distinct sampling strategies based on this comparative analysis. If the measured distance within a given patch is less than $D_{th}$ , it indicates a lower complexity of features. Consequently, the centroids of ten identified clusters from the patch are selected as the seed points for the sampling process. The extraction approach in this scenario employs the minimax facility location greedy core-set selection [27] technique at a sampling rate of 10%. This conservative sampling rate is predicated on the lower feature density.

Alternatively, should the distance surpass the threshold $D_{th}$ , it signifies a greater complexity of features within the patch. To accommodate the richer feature set, we revise the cluster count to K = 20 and execute fresh clustering. The centroids of these augmented clusters then serve as the initial samples for an increased sampling rate of 20%. This methodological adjustment ensures denser retention of feature vectors in areas abundant with feature-related information, effectively addressing sampling biases between patches of contrasting feature densities. Consequently, this allows for an optimized usage of memory resources, ensuring the retention of salient details and contributing positively to the robustness of the feature representation.

By adapting the sampling rate to the relative feature richness of the patches, this refined approach ensures a judicious allocation of memory, enabling the conservation of a more significant quantity of useful information. This sampling strategy effectively balances thorough feature capture with efficient memory use, enhancing the efficacy of anomaly detection systems.

### 3.4. Anomaly Detection

**Figure 5:**
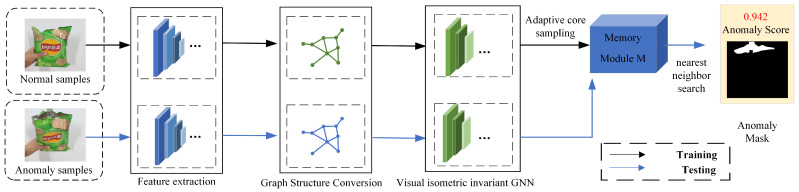
Overall defect detection process.

Figure 5 below shows the complete flowchart of the training and testing process for our model. It roughly demonstrates the process of extracting features from normal samples to construct the memory module, as well as how test samples have their features extracted and then queried against the features in the memory module. During the testing phase, the procedure commences with the target image being introduced into an ImageNet pre-trained network. This initial step facilitates the extraction of two distinct types of features: shallow features, which capture basic image attributes, and deep semantic features that encapsulate higher-level contextual information. Following this dual feature extraction, the next phase involves the fusion of these attributes to derive rotationally invariant features through the use of GCN [24].

Post-feature fusion, the ASPM computes the features for each image patch, enabling a comprehensive feature representation. This process is akin to the PatchCore algorithm, where each patch’s features are methodically compared against a corresponding memory bank to ascertain the data point exhibiting the maximum distance. This identified distance then forms the basis for calculating the anomaly score, which serves as a predictive measure for the presence of anomalous patterns within the patch.

Once the anomaly scores for all patches are determined, the results undergo upsampling via bilinear interpolation to align with the original image resolution, thereby ensuring consistency with the initial input dimensions. Additionally, the results are refined through the application of a Gaussian filter with a kernel width of σ = 4 [3]. This smoothing operation leverages the Gaussian distribution’s properties to mitigate noise and artifacts, thereby leading to a more coherent and visually palatable representation of anomaly scores.

The utilization of advanced neural network architectures and sophisticated techniques, including GCN and ASPM, in conjunction with preprocessing and post-processing refinement steps embodies the rigor and precision that are characteristic of such methodologies. These careful considerations in feature extraction and result optimization contribute to the robustness and accuracy of the anomaly detection process.

## 4. Experimental Results and Analysis

### 4.1. Experimental Details

We conducted a series of experiments to evaluate the performance of our model. In the comparison experiments, we visualize ASPM’s ability to localize anomalies by the value of AUROC. In the small-sample experiments, we verify the accuracy of the model for small sample sizes such as 1,2,4, and 8. In the ablation experiments, we reflect the contribution of different parts of the model to the model performance by using them separately.

The experimental setup for our study consists of the following technical specifications and platforms: The operating system deployed was Windows 10. Hardware components included an NVIDIA RTX3070 graphics card and an Intel^®^ Core™ i5-12490F processor. The network architecture for this research was constructed utilizing the PyTorch 1.8 deep learning framework, accompanied with Python version 3.7.4 as the programming language.

Our investigations were performed on the MVTec AD dataset [28], a benchmark that includes 3629 training images and 1725 testing images spanning 15 different industrial categories. This dataset is separated into two segments: 10 object categories and 5 texture categories. During preprocessing, we applied center cropping and standard normalization procedures to the images. The processed images were set to a uniform dimension of 224 × 224 pixels. Subsequently, we divided each image into N = $N_{p}$ = 49 patches. Feature extraction was carried out using the initial three layers of the Wide-ResNet50 [25]—a practice that is consistent with methodologies employed in PatchCore [3] and PaDiM [20]. Memory vectors were downsampled at an initial rate of 10% with parameter D set to 3. During the inference stage, we selected the four memory vectors closest to each target vector for analysis.

Further testing was conducted on a custom dataset tailored to identify breakages in household goods, a dataset assembled by our team. This proprietary dataset is categorized into three types of merchandise: bottles, bags, and cans. In each category, we collected 200 images of products in normal condition and 40 images of products with packaging damage, all sourced from real retail environments. The images were standardized to 224 × 224 pixels in size. For evaluation purposes, the anomaly detection performance was benchmarked using the area under the receiver operating characteristic (AUROC), measuring anomaly localization accuracy at both image and pixel levels. AUROC serves as a principal metric in most image anomaly detection studies, providing an insightful gauge for model performance. The area under the ROC (receiver operator characteristic curve) means the larger the AUROC, the better the model performance.

Moreover, we assessed the inference speed of the model based on frames per second (FPS), offering a quantitative measure of the model’s operation tempo. These evaluations are critical for ensuring that our proposed model adheres to the high standards of performance and reliability expected within the scientific community.

### 4.2. Comparative Experiments

**Figure 6:**
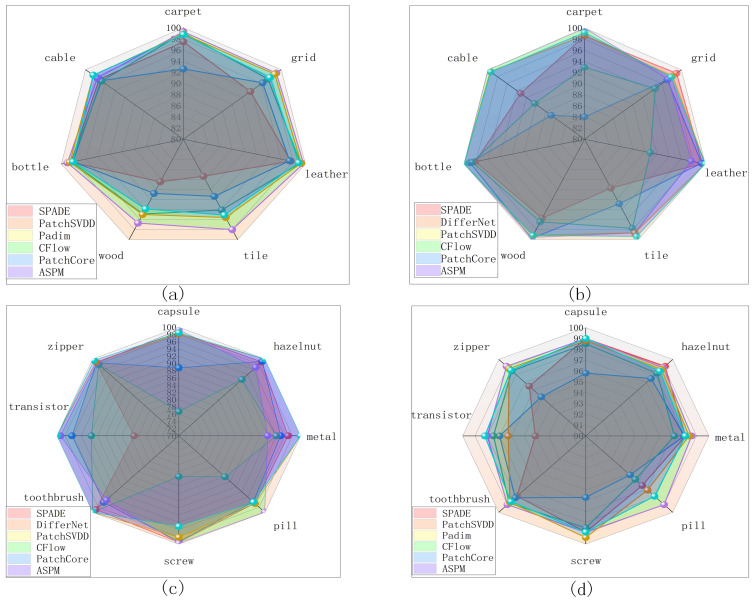

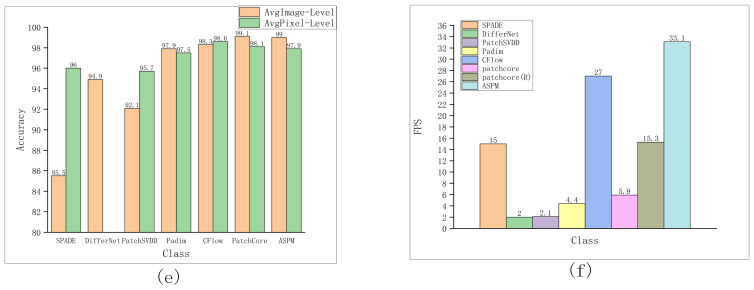
Multi-model performance comparison. (**a**) is a comparison of image-level AUROC performance across seven models for seven categories including carpet. (**b**) is a comparison of pixel-level AUROC performance across seven models for seven categories including carpet. (**c**) is a comparison of image-level AUROC performance across seven models for eight categories including capsule. (**d**) is a comparison of pixel-level AUROC performance across seven models for eight categories including capsule. (**e**) is a comparison of the average image-level and pixel-level AUROC performance across seven models on the MVTec AD dataset. (**f**) is a comparison of FPS (frames per second) performance across seven models on the MVTec AD dataset.

Figure 6 presents a comprehensive analysis of the performance of six different models tested on the MVTec Anomaly Detection dataset [28]. Figure 6a–d reflect the performance of different models for defect localization at their level by their AUROC values at the image and pixel level; Figure 6e reflects the overall performance of the models for defect localization performance by the average values of AUROC for different classes; and Figure 6f reflects the speed of these models by frames per second (FPS). Figure 6a,c illustrate the efficacy of the models at the image level, whereas Figure 6b,d delineate the models’ performance at the pixel level. Each model is uniquely identified by a distinct color within these representations. The size of the area occupied by the different colors indicates the level of localization performance of the model—the larger the area, the more proficient the model. The purple color represented by the ASPM covers a large portion of the area in many test classes.

The empirical results spotlight ASPM’s superior speed, outpacing the methods referenced in [3,12,14,16,20,29]. Within our system configuration, ASPM performs nearly twice as swiftly as PatchCore, achieving a formidable throughput of 33.1 frames per second, thereby demonstrating a real-time capability in anomaly detection.

Furthermore, Figure 6a–e show that our ASPM method not only outperforms our competitors in terms of speed but also achieves detection and segmentation performances on par with those of current state-of-the-art models and even sets benchmarks in terms of detection and segmentation performances in several specific categories (particularly transistors and zippers).

This multifaceted evaluation, reflecting both high accuracy and exceptional processing speed, underscores the ASPM’s potential as a leading-edge solution in the realm of anomaly detection within industrial imaging scenarios.

### 4.3. Small Sample Experiments

To validate the training performance of our model on small samples, we performed an experiment using PatchCore and GraphCore. We use the same approach [9] to validate the performance of the model for defect detection of rotating goods with fewer samples. We denote by Aug.(R) the data augmentation (rotation) using PatchCore, where we augment the data (e.g., by rotating the data) before extracting features from the pre-trained model from ImageNet. In the small-sample learning condition, we set the number of training samples n to 1, 2, 4, and 8. PatchCore, which does not use data augmentation, is compared with our model. Table 1 shows the differences in framework between the different models.

**Figure 7:**
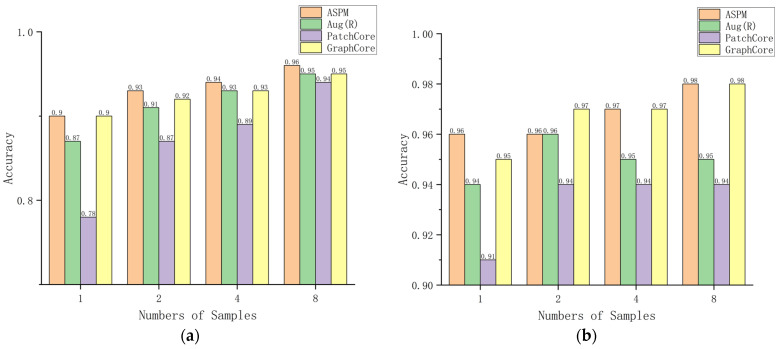
(**a**) The image-level AUROC of ASPM, Patchcore Aug(R), and GraphCore with sample sizes of 1, 2, 4, and 8, while (**b**) shows the pixel-level AUROC for the same cases. Together, the two figures reflect the performance of the models.

The experimental findings corroborate our initial hypothesis. As depicted in Figure 7, the introduction of data augmentation techniques to PatchCore significantly bolsters its memory repository by infusing it with rotated image features. This enrichment results in a tangible improvement in the model’s accuracy for both image-level and pixel-level anomaly detection by 1 percentage point for *n* = 8, compared to the baseline PatchCore performance.

In contrast, our proposed model circumvents the necessity for data augmentation altogether. By adeptly extracting rotated features through the use of Graph Convolutional Networks (GCN) [24], our model not only matches but surpasses the augmented PatchCore (denoted as Aug.(R)) by registering a 1 percentage point higher accuracy at both image and pixel levels for *n* = 8. This enhanced accuracy is even more appreciable for *n* ≤ 8.

When compared with GraphCore, it has been observed that the model accuracy of our system is nearly identical at the pixel level. However, when evaluating at the image level, our model demonstrates marginally superior performance with an increase of 0.1 percentage points in accuracy over GraphCore, consistent across sample sizes of 2, 4, and 8.

The data underscore the efficacy of leveraging CNN+GCN for feature extraction, which imbues our model with the innate proficiency to excel in anomaly detection tasks without the added complexity of external data manipulation. This characteristic significantly simplifies the process and reiterates the robustness of our model, positioning it favorably for scientific and industrial applications where efficiency and accuracy are paramount.

### 4.4. Ablation Experiment

Table 2 elucidates the efficacy of our method in enhancing both anomaly detection and localization. Four distinct experimental scenarios showcase the versatility of our approach:

- (A) PatchCore-10% is executed on our proprietary hardware configuration;
- (B) Leverages a patch-wise memory schema exclusively;
- (C) Combines patch-wise memory with standard coreset sampling;
- (D) Entails full-frame processing underpinned by adaptive coreset sampling complemented with Grad-CAM utilization.

The findings presented in Table 2, particularly within experiments (B), (C), and (D), affirm that partitioning the memory architecture on a patch-wise basis significantly streamlines the inference timeline. These tests were conducted using GPU-accelerated systems, which indicate marginal improvements when adaptive subsampling is introduced. However, these benefits are posited to be markedly accentuated in practical, real-world applications.

Experiment (C) in Table 2 delineates how employing patch-wise adaptive sampling contributes to a 0.6 percentage point increase in image anomaly detection accuracy and a 0.2 percentage point increase in segmentation performance—all this while managing to retain processing alacrity.

Moreover, as demonstrated in Table 2(D), the application of Grad-CAM to mitigate background noise has an observable, albeit slight, deceleration effect on computation speed. Nevertheless, this is counterbalanced by a consequential improvement in accuracy metrics. These findings distinctly suggest that the strategic application of adaptive sampling methods and attention-focused mechanisms, such as Grad-CAM, can yield substantial advancements in the domain of anomaly detection, not solely in algorithmic precision but also in operational efficiency.

### 4.5. Visualization of Results

**Figure 8:**
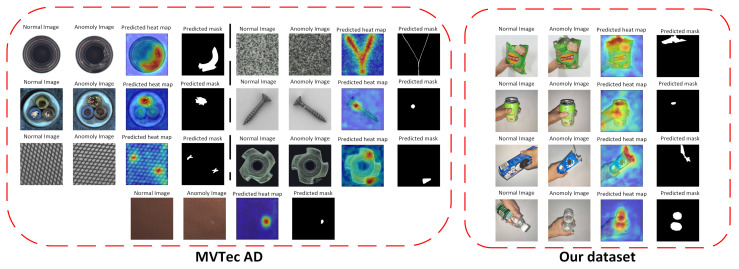
Visualization of experimental results.

Figure 8 presents the visualization outcomes from applying our method to the MVTec Anomaly Detection (AD) dataset as well as a custom-built dataset for detecting breakages in goods, with the training regime involving four samples per category. In each batch of images, the sequence from left to right includes a normal image, a detection result, an anomaly score map, and the ground truth annotation.

The visual evidence suggests that our method achieves commendable performance in localizing anomalies across a diverse array of object types, denoting a high level of robustness intrinsic to the system. The results underscore the method’s capabilities in few-shot learning scenarios, as it demonstrates proficiency in a four-shot context. The method’s effectiveness in such limited-data environments illustrates its potential practicality for real-world applications where collecting extensive amounts of annotated data may be challenging or infeasible.

## 5. Discussion and Conclusions

In the present study, we introduce a novel algorithm devised to enhance breakage detection in rotated goods, with pivotal findings summarized as follows:

1. The synergy of graph convolutional networks (GCN) and convolutional neural networks (CNN) is harnessed to distill rotation-invariant features, effectively addressing the challenge posed by varied orientations of goods;
2. The application of Grad-CAM adeptly mitigates the effects of complex environmental factors on defect detection, demonstrating a refined approach to discerning anomalies in intricate settings;
3. We propose the ASPM, an innovative construct that dynamically modulates the volume of subsamples through boundary distance computations of feature vectors. This approach judiciously curtails the embedding of superfluous upsampled elements, thereby diminishing computational overhead and memory demands.

The research elucidates the strengths of using GCN to procure rotationally invariant features within isomorphic datasets. Moreover, the ASPM framework paves the way for cultivating high-performance models utilizing a minimal collection of normal samples, which serves as a cornerstone for augmenting the accuracy of defect detection in small-sample scenarios.

Projected future endeavors aim to refine the detection efficacy for shape-variable objects, including those encapsulated in flexible packaging, by continuing to evolve the algorithmic approach detailed herein. These refinements are critical stepping stones in advancing the field of automated visual inspection.

## Figures and Tables

**Table 1:** Comparison of three feature extraction network structures.

Data Augmentation	Feature Extraction Module Structure	Name
None	ImageNet Pre-trained Model	PatchCore
Rotation	ImageNet Pre-trained Model	Aug.(R)
None	GNN	GraphCore
None	ImageNet Pre-trained Model+GNN+Grad-CAM	ASPM

**Table 2:** Comparison of ablation test performance.

	Grad-CAM	Patch- Wise	Adaptive Sampling	Img AUROC	Pixel AUROC	FPS
A				98.9	98.1	13.4
B		√		98.5	97.5	24.1
C		√	√	99.0	98.0	33.1
D	√	√	√	99.1	98.2	32.9

## Data Availability

No new data were created or analyzed in this study. Data sharing is not applicable to this article.
